# Electrophysiological Correlates of an Alcohol-Cued Go/NoGo Task: A Dual-Process Approach to Binge Drinking in University Students

**DOI:** 10.3390/ijerph16224550

**Published:** 2019-11-18

**Authors:** Javier Blanco-Ramos, Fernando Cadaveira, Rocío Folgueira-Ares, Montserrat Corral, Socorro Rodríguez Holguín

**Affiliations:** Department of Clinical Psychology and Psychobiology, Universidade de Santiago de Compostela, 15782 Santiago de Compostela, Spain; fernando.cadaveira@usc.es (F.C.); rocio.folgueira@usc.es (R.F.-A.); montse.corral@usc.es (M.C.); rodriguez.holguin@usc.es (S.R.H.)

**Keywords:** alcohol consumption, binge drinking, adolescence, dual-process model, response inhibition, neurocognitive, event-related potentials, Go/NoGo

## Abstract

Binge drinking is a common pattern of alcohol consumption in adolescence and youth. Neurocognitive dual-process models attribute substance use disorders and risk behaviours during adolescence to an imbalance between an overactivated affective-automatic system (involved in motivational and affective processing) and a reflective system (involved in cognitive inhibitory control). The aim of the present study was to investigate at the electrophysiological level the degree to which the motivational value of alcohol-related stimuli modulates the inhibition of a prepotent response in binge drinkers. First-year university students (*n* = 151, 54 % females) classified as binge drinkers (*n* = 71, ≥6 binge drinking episodes, defined as 5/7 standard drinks per occasion in the last 180 days) and controls (*n* = 80, <6 binge drinking episodes in the last 180 days) performed a beverage Go/NoGo task (pictures of alcoholic and nonalcoholic drinks were presented according to the condition as Go or NoGo stimuli; Go probability = 0.75) during event-related potential recording. In binge drinkers but not controls, the amplitude of the anterior N2-NoGo was larger in response to nonalcohol than in response to alcohol pictures. No behavioural difference in task performance was observed. In terms of dual-process models, binge drinkers may require increased activation to monitor conflict in order to compensate for overactivation of the affective-automatic system caused by alcohol-related bias.

## 1. Introduction

Alcohol is one of the oldest and most common recreational substances consumed worldwide as part of many cultural and social practices, providing perceived pleasure to many of its users. The negative consequences of alcohol consumption are a major public health concern, as reflected in the World Health Organization’s global status report on alcohol and health [1]. One of the main issues addressed in this report is alcohol consumption in adolescents and young adults. More than a quarter (26.5%) of all 15–19 year olds worldwide (i.e., 155 million adolescents) are current drinkers. Specifically, binge drinking (BD), a pattern of alcohol consumption that brings the blood alcohol concentration (BAC) to 0.08 g/dl or above [2], is the most common pattern of consumption among the young population in Western societies, peaking at age 20–24 years [3]. This pattern of drinking is most widespread during university years, with a subsequent decline in alcohol use once adult responsibilities prevail [4]. Despite the general tendency for this pattern of consumption to be abandoned after university, binge drinking has been recognized as a risk factor for alcohol use disorders (AUDs) during adulthood [5].

### 1.1. Alcohol Use Disorders and Dual-Process Models

From a neurocognitive perspective, drug addictions (e.g., AUDs) have been explained by dual-process models as a loss of willpower to resist the drugs [6]. Specifically, impaired decision-making abilities have been described in addictive disorders, as individuals are unable to make choices based on long-term outcomes when confronted with short-term benefits. According to dual-process models, these abilities result from a balance between two distinct but interacting systems [7]: (a) a slow reflective system, involved in deliberate responses through memory and executive functions, and (b) an affective-automatic system, involved in the emotional evaluation of stimuli and appetitive responses. The imbalance between these two systems has been proposed as the basis for the perpetuation of alcohol use disorders and addictions by means of a hyperactive affective-automatic system linked to increased motivational qualities of the drug (i.e., alcohol) as well as an impaired reflective system (including cognitive inhibitory control) that fails to control short-term urges [8]. Although these models have received criticism due to oversimplifications [9,10], they are under continuous revision and provide a more accurate account of adolescent risk taking than prior models that have mainly focused on cognitive deficiencies [11].

### 1.2. Adolescence and Dual-Process Models

Dual-process models have largely been validated in alcohol dependence and have been suggested to be an explanatory framework for risky behaviour, such as binge drinking, in adolescents [12]. A cornerstone of these models posits that adolescence is characterised by a neurodevelopmental delay of the reflective system relative to the affective-automatic system [13], which may lead to more impulsive decision making and immediate reward-seeking behaviour [14].

Balance between the two systems is not reached until emerging adulthood, when, under normal conditions, executive functions mature [15]. As a consequence, higher rates of substance experimentation and misuse are reported during this stage of development, including alcohol consumption [16], which is often correlated with other types of undesirable behaviour, such as increased rates of violence, unsafe sexual relations, and dangerous driving [17,18,19]. Considering that the neurotoxic effects of alcohol are especially deleterious to developmental stages such as adolescence (as seen in animal studies, e.g., Reference [20]), the establishment of a binge drinking pattern of consumption at this stage might disrupt maturation of the reflective system. Persistence of an imbalance between the reflective and affective systems could lead to substance use disorders and other behavioural dysregulations [8]. In this respect, the development of alcohol-related bias, whereby alcohol cues become more important than other stimuli, may contribute to the perpetuation of alcohol use disorders [21].

### 1.3. Effects of Binge Drinking on the Reflective and Affective Systems

Until now, most neurocognitive research on the deleterious effects of binge drinking in adolescents and young adults has only focused on the reflective system and less is known about affective and motivational disturbances or how the two systems interact [12].

Regarding the reflective system, numerous studies have used neuropsychological, electrophysiological, and neuroimaging approaches to address cognitive alterations related to binge drinking. At the neuropsychological level, a systematic review [22] has shown that binge drinking is associated with deficits in verbal memory and executive functions, with focus on subcomponents of cognitive inhibitory control. Specifically, deficits were found to be related to response inhibition (the ability to suppress prepotent or inappropriate responses), interference control (the ability to inhibit prepotent mental representations), and self-control (which involves resisting temptations and not acting prematurely). Furthermore, binge drinkers (BDs) may display impairments in prospective memory, cognitive flexibility, and self-monitoring of information in working memory. Regarding decision-making abilities, on which dual-process models focus, some findings indicate poor decision making in young BDs as a result of hypersensitivity to reward. However, the authors of such studies have highlighted the need for further research to overcome inconsistences between study findings, particularly those related to decision-making abilities.

To date, some reviews have already encompassed the main findings on both neuroimaging and electrophysiological data exploring binge drinking correlates [23,24,25]. The advantage of these techniques is that they enable the detection of alterations at the neural level even in the absence of differences in behavioural performance. Electrophysiological research has detected alterations in event-related potentials (ERPs) and oscillatory activity of BDs related to cognitive processes such as attention, working memory, associative memory, and response inhibitory control. Functional magnetic resonance imaging (fMRI) studies have reported abnormal brain activity in BDs during tasks involving verbal learning, working memory, and response inhibition. Although some specific findings of different studies are not consistent, these reviews note that cognitive alterations are definitely related to binge drinking in adolescents and young adults.

Research on affective and motivational systems in binge drinking is scarce. Electrophysiological studies have reported alterations in ERP components related to emotional processing [26] and categorization [27] of affective human voices. A recent study [28] found alterations related to crossmodal integration of emotional stimuli in perceptual (P1) and decisional (P3b) ERP components in young BDs. Furthermore, when processing alcohol-related stimuli, strong alcohol-related biases have been observed in BDs [29,30]. Due to their motivational value, alcohol-related cues seem to increase brain activation related to attentional processes and emotional evaluation of stimuli in BDs [31,32,33].

In summary, most research on the neurocognitive consequences of binge drinking in adolescents and young people has explored both systems of the dual-process models (i.e., reflective and affective-automatic) by including each separately, even though they act interactively and are therefore intrinsically related [11,12]. Given that the main model assumption refers to an imbalance between the two systems, research studies should try to consider the whole picture. Only some recent paradigms have fulfilled this criterion. Specifically, Go/NoGo tasks including stimuli with motivational value (i.e., alcohol-related pictures) represent the most employed paradigm.

### 1.4. The Go/NoGo Paradigm.

Go/NoGo tasks require participants to respond quickly and accurately to a typically frequent signal (Go stimuli) so that a prepotent tendency to respond is created. In a less frequent subset of trials (e.g., 25%), a different signal (NoGo stimuli) is presented and the response should be withheld. As a result, Go/NoGo tasks challenge the subject’s ability to withhold a prepotent response and NoGo trials are thus associated with an increased rate of false alarms [34]. At the electrophysiological level, two event-related potential (ERP) components have consistently been identified in relation to NoGo trials with visual tasks, namely N2-NoGo and P3-NoGo [35].

The first component, N2-NoGo, comprises a negative shift that peaks between 200 and 300 ms at midline frontal electrodes. The neural sources of this component have been located in the inferior frontal cortex (IFC) [36,37] and anterior cingulate cortex (ACC) [38,39]. Although the functional meaning of N2 is still a matter of debate, recent and cumulative findings depict it as an index of detection of conflict between competing responses (prepotent Go vs. NoGo) and posterior adjustment [36,40,41].

The second major ERP component, P3-NoGo, is a positive wave that peaks between 300 and 600 ms at frontocentral or parietal locations [42]. The neural source of P3-NoGo has been located in the IFC [36]. Regarding its functional meaning, P3-NoGo may reflect the evaluation or even closure of the response inhibition process [43].

### 1.5. Tasks with Alcohol-Related Cues and Binge Drinking

The addition of motivationally salient alcohol-related stimuli in the Go/NoGo paradigm (as a Go and/or NoGo signals, as a cue, or as background of a neutral Go/NoGo task) has provided a strategy for exploring the two systems postulated by the dual-process models in relation to binge drinking. More importantly, the joint exploration of both systems in the same task addresses recent claims emphasizing that both systems are intrinsically related or even represent overlapping elements of a continuous unitary system [44].

At the behavioural level, in a speeded Go/NoGo task using pictures of alcoholic drinks, neutral pictures and pictures of soft drinks as Go and NoGo targets, BDs displayed poorer performance-monitoring abilities. Specifically, BDs showed difficulty in adjusting after errors, especially when these errors involved alcohol-related cues [45]. In another study, in a comparison of the performance of alcohol and neutral (geometrical shapes) Go/NoGo tasks, BDs (in contrast to non-BDs) displayed an alcohol-specific impairment of response inhibition [46].

At the electrophysiological level, a very similar task design showed that participants who became intoxicated more frequently exhibited an enhanced N2 in response to alcoholic stimuli relative to individuals who became intoxicated less often [47]. When alcohol vs. neutral pictures were used as a background of a letter Go/NoGo task, BDs showed delayed P3 latencies, together with higher commission errors regarding the alcohol pictures relative to the neutral pictures [48]. Furthermore, reduced N2 amplitudes were found in BDs in a beverage Go/NoGo task when a response to alcohol pictures had to be withheld, relative to the neutral condition. These alterations did not emerge when task instructions switched to Go/NoGo sparkling vs. non-sparkling beverage pictures [49]. In contrast, in subjects with low sensitivity to alcohol (with a pattern of consumption similar to BDs) who completed an alcohol cued Go/NoGo task, N2 and P3 amplitudes were larger during low probability NoGo trials cued by an alcohol-related image [50].

One fMRI study [51] used an alcohol version of the Go/NoGo task in young, light and heavy drinkers. Heavy drinkers showed greater activation in the right dorsolateral prefrontal cortex, anterior and midcingulate cortex, and right anterior insula during NoGo alcohol trials; the authors proposed that the presence of attentional bias to alcohol-related cues (due to its increased incentive value) in heavy drinkers resulted in the need for greater neural activation to withhold a prepotent response.

In conclusion, research findings on alcohol-related Go/NoGo tasks suggest that BDs may present neurocognitive alterations related to inhibition of a prepotent response, which may be modulated by the motivational value of the stimuli (i.e., alcohol pictures). However, these studies indicate different processes related to Go/NoGo tasks (conflict monitoring, evaluation of response inhibition, etc.). Although most of these findings are compatible, the direction is not fully consistent. Some of the research described above did not find group differences in behavioural task performance, while others did observe such differences [46,48]. BDs showed increased N2 ERPs amplitudes or fMRI activations, although some studies found no differences [48] or a decrease on this ERP component [49]. The need for further investigation is a commonly highlighted request in all of the studies reviewed.

Furthermore, the inclusion of alcohol-related cues in attentional paradigms [52,53] showed that a repeated pattern of alcohol consumption may cause alcohol-related stimuli to induce a conditioned appetitive value, which will trigger the motivational system. Early attentional bias to alcohol cues was observed in subjects with a binge drinking pattern of consumption and was indicated by increased P1 amplitude. In visual paradigms, this component describes a positive wave that peaks at around 100–130 ms at lateral occipital sites. In relation to perceptual processing, P1 is sensitive to the physical characteristics of the stimuli and attentional influences [54].

### 1.6. Aims of the Study

In order to shed some light on this approach, we aimed to examine at the electrophysiological level the degree to which the motivational value of stimuli modulates the inhibition of a prepotent response in BD subjects. An alcohol-related Go/NoGo task was designed for this purpose. In the context of dual-process models, the use of alcohol (Al) and nonalcohol (NoAl) pictures as Go or NoGo stimuli allowed us to encompass the interaction between the reflective (i.e., inhibitory control of a prepotent response) and the affective-automatic systems (i.e., attentional bias to motivationally appetitive stimuli). The initial working hypothesis was that conflict monitoring and/or inhibitory difficulties will be greater in BDs than in control (CN) subjects, particularly in the presence of alcohol-related stimuli. In terms of ERPs and on the basis of previous findings [47,50], we expected the amplitudes of those components associated with conflict monitoring and response inhibition (N2-NoGo and P3-NoGo) to be larger in BDs than in controls. Furthermore, according to previous findings [52,53], we hypothesized that Al stimuli will be more salient and that this will be reflected in a larger amplitude of the early P1 component in BDs than in controls.

Finally, we aimed to explore possible gender/sex differences in relation to our hypothesis. To our knowledge, no previous research on binge drinking has reported gender/sex modulations in Go/NoGo tasks, independently of the use of alcohol or neutral stimuli. Some studies specifically did not find gender/sex-related effects [50,55], while others have reported nonsignificant gender tendencies [46]. In many cases, gender/sex was not explored due to small sample sizes. In other ERP studies including alcohol-related cues, occasional gender-related modulations were reported (e.g., in an oddball paradigm [33] and in a stop signal task [56]). We therefore wished to explore this issue further.

## 2. Materials and Methods

### 2.1. Sample Selection

The final sample in this study comprised 151 participants (ages 18–19), selected within the framework of broader research on epidemiological, neurocognitive, and academic consequences of binge drinking among university students. For sample selection, a total of 2998 first-year students from the University of Santiago de Compostela (Spain) voluntarily and anonymously completed a questionnaire assessing sociodemographic information and alcohol and other substance consumption, including the adapted versions of the Alcohol Use Disorders Identification Test (AUDIT) [57,58], the Cannabis Abuse Screening Test (CAST) [59,60], and the Nicotine Dependence Syndrome Scale, short version (NDSS-S) [61,62]. AUDIT-C scores were considered for preselection of binge drinking (BD) and control (CN) groups (over and under 3/4 for men and women, respectively).

The following preselection criteria were applied to the classroom questionnaire: (1) provision of contact information (phone number and e-mail) as a sign of willingness to enter subsequent phases of the study, (2) 18–19 years old, and (3) nonconsumption of illegal drugs except cannabis. The 2998 completed questionnaires yielded 516 subjects who met these criteria and agreed to participate in the study. These subjects then completed a semi-structured interview and a set of questionnaires, including medical history, the Alcohol Timeline Follow-back (TFLB) (180 days), the Cannabis TLFB (90 days; only to subjects who reported cannabis consumption at some time throughout their lives) [63], the Spanish versions of Symptom Checklist-90-Revised (SCL-90-R) [64], and the Barrat Impulsivity Scale (BIS-11) [65]. Those interviewees who met the inclusion/exclusion criteria listed in Table 1 were considered for enrolment in the event-related potentials (ERP) phase of the study.

All participants gave written informed consent and received monetary compensation (20 euros) for their participation (interview and ERP assessment). The study was approved by the Bioethics Committee of the University de Santiago de Compostela.

Of the 159 subjects who met the inclusion criteria and completed the ERP assessment, 8 subjects were excluded due to the poor quality of the ERP recordings in the Go/NoGo task. Specifically, 3 subjects were rejected due to poor behavioural performance (more than 3 SD below the mean) and 5 subjects because of EEG artefacts (resulting in less than 60% or 20 EEG segments preserved for averaging). The final sample comprised 151 subjects: 80 were assigned to the control (CN) group (38 females) and 71 to the binge drinking (BD) group (43 females). The sociodemographic and alcohol/drug consumption data for each group are summarized in Table 2

### 2.2. Task and Procedure

Participants were asked questions about their physiological/health conditions when they arrived at the laboratory. Only those participants who had slept for at least 6 h the night before, were free of diseases, and had not taken any medication in the last 3 days and who had not consumed alcohol or cannabis in the previous 24 h or tobacco or stimulating drinks in the previous 3 h undertook the tasks. None of the subjects were excluded because of these demands, although one participant had to schedule a new appointment. A breathalyser test was administered to verify a 0.00% breath alcohol level.

**Figure 1:**
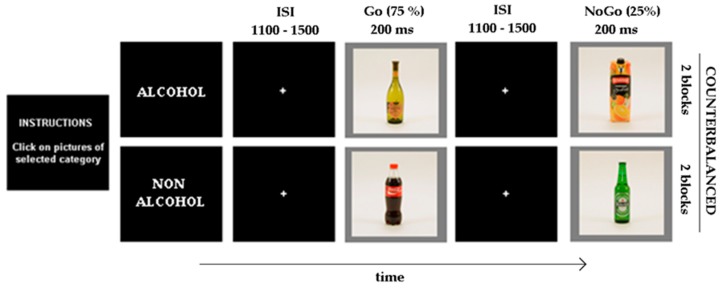
Beverage Go/NoGo task: Subjects were instructed to respond only to the Go stimuli (Alcoholic -Al- or nonalcoholic -NoAl- beverages according to the condition) by pressing the left-hand button of the mouse.

During EEG recording, each subject sat on an armchair inside an electrically shielded, dimly lit, sound-attenuated room. Subjects were instructed to avoid making voluntary movements during EEG recording and to fix their gaze on a small cross in the centre of the screen. The task consisted of a Go/NoGo paradigm (Figure 1) with images of beverage as stimuli and two task conditions.

After general instructions were given, the Go matching category (alcoholic or nonalcoholic) was displayed on the screen. In the alcohol condition (Go-Al), pictures of alcoholic (Al) drinks (wine, beer, or liquor) were defined as Go stimuli and pictures of nonalcoholic (NoAl) drinks (water, soft drinks, and milk) were defined as NoGo stimuli. The assignation was reversed in the nonalcohol (Go-NoAl) condition. Subjects were required to respond as quickly and accurately as possible to Go stimuli (by pressing the left-hand button on the mouse with the dominant hand) and to omit any response to NoGo stimuli. A brief practice block was run before the recording to ensure that participants understood the instructions and were familiar with the task. The picture set was designed to include drinks representative of Spanish consumption habits and represented both active (people drinking) and passive (bottle on a tray) images, following the same criteria as in the Amsterdam Beverage Picture Set (ABPS) [69].

The stimuli (500 × 500 pixels over a grey background, 200 ms duration) were randomly presented in the centre of a cathodic ray tube monitor (resolution 1152 × 864 ppp, refresh rate 85 Hz) at a distance of 100 cm, with an 1100–1500-ms interstimulus interval; a fixation cross was presented in the centre of the screen during the interstimulus interval. Each condition (Go-Al vs. Go-NoAl) comprised 192 stimuli (25% NoGo) divided into two consecutive blocks (each 2.5 min in duration) with a brief pause between them. The order of the conditions was balanced among subjects (47.5 % of the CN and 56.3 % of the BD subjects received the Go-Al condition first: chi square test, no significant difference).

### 2.3. EEG Recording and Processing

The electroencephalogram (EEG) was recorded using an Acticap system (Brain Products, Munich, Germany) with 64 electrodes located according to the extended 10–20 International System [70]. The ground electrode was placed at Fpz and a reference electrode was placed on the nose tip. Impedances were kept below 20 kΩ. The EEG signal was amplified with BrainAmp DC amplifiers and filtered with a band-pass filter of 0.01–100 Hz and a 50-Hz notch filter. Sampling rate was 500 points/s. Eye movements and blinks were recorded via bipolar horizontal (HEOG) and vertical (VEOG) channels.

EEG data were processed with BrainVision Analyzer software (Version 2.1) (Brain Products GmbH, Scientific Support, Gilching, Germany). The method proposed by Gratton, Coles, and Donchin [71] was applied to correct ocular artefacts. In a small number of cases (less than 7%) in which this method was not satisfactory (determined by visual inspection), independent component analysis (ICA) was applied [72]. Data were digitally filtered offline with a 0.1–30 Hz band-pass filter. Stimulus-locked EEG epochs of 1000 ms were created, with a pre-stimulus period of 100 ms. Baseline was adjusted to 0 µV, and epochs with artefacts exceeding ±80 µV were rejected. Epochs were averaged according to four categories (Go-Al, Go-NoAl, NoGo-Al, and NoGo-NoAl). Only those epochs associated with stimuli with a correctly executed response (Go stimuli followed by a correct response between 100 and 1000 ms; NoGo stimuli followed by no response) were included.

### 2.4. Principal Components Analysis

Averaged data epochs for each category were exported from BrainVision Analyzer and then examined by principal component analysis with Dien’s ERP PCA toolkit (v. 2.68) [73] in MATLAB (The Mathworks, v. 9.4.0.885841, R2018a, Natick, MA, USA). Data were resampled to 250 points/s. Temporal principal component analysis was then computed with promax rotation. Parallel testing [74] resulted in selection of 10 factors, of which the peak latencies, polarity, and explained variance are summarized in Table 3.

**Figure 2:**
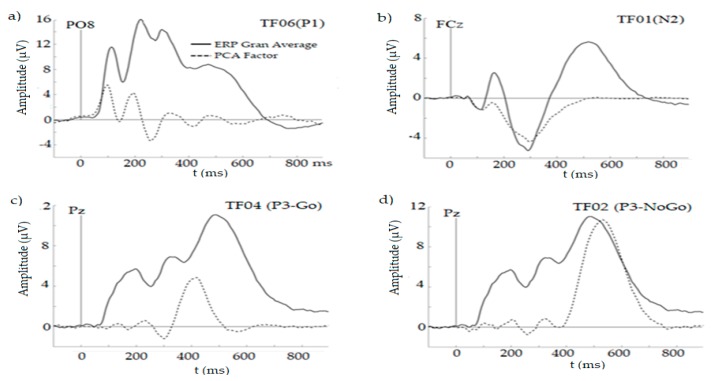
Graphical plots of the main ERP components identified: (**a**) TF01, P1; (**b**) TF01, N2, (**c**) TF04, P3-Go, (**d**) TF02, P3-NoGo, Grand average (solid line) versus PCA factor (dashed line) is shown for each component at the location of the maximum peak.

Each component extracted was plotted as a time-course waveform rescaled to microvolts by multiplying the correlation factor loadings by the standard deviations of the variables to produce covariance loadings [75]. The factors and their plots were examined to identify corresponding ERP waves (last column of Table 3), which are illustrated in Figure 2.

The factor values were rescaled to µV and used for further analyses. Mean voltage values on a ±12 ms window around peak latency of each component of interest (according to our hypothesis, P1, N2, P3-Go, and P3-NoGo) were extracted, for each subject, condition, and type of stimulus, averaging five electrode locations (at the site of maximum amplitude and the four surrounding channels). Values greater than mean ± 3 standard deviations for each condition and type of stimulus were considered outliers and were replaced by average values. A low number of outliers was found for each factor: 2 cases each for N2, P3-NoGo, and P3-Go and 4 cases for P1.

### 2.5. Statistical Analysis

Both ERP and behavioural data were statistically analysed (IBM Statistical Package for Social Sciences (SPSS), v.23). Preliminary analyses were conducted following the recommendation of Joel and Fausto–Sterling [76] for studies with both females and males as subjects. According to these authors, the sex variable should only be retained in the analyses when an interaction with the main variable in the research (i.e., group) reaches a significant level. As a result, the sex variable was excluded from all ERP analyses except for P3-NoGo, for which a significant group × sex × type of stimulus (described below) was observed. Furthermore, although regular cannabis consumers were excluded, we found that residual cannabis consumption in the last 3 months was imbalanced between groups; therefore, this measure was included as covariate to control potential confounding effects.

Regarding the ERP data, after verifying the assumptions of parametric tests, mixed-model analyses of covariance (ANCOVAs) were computed according to the hypotheses, as follows: (1) In order to test the expected larger amplitudes of ERP components associated with conflict monitoring and response inhibition in BDs, only NoGo trials were analysed. For P3-NoGo, we applied a 2 × 2 × 2 design, including group (BDs vs. CNs) and sex (male vs. female) as the between subject factor and type of stimuli (Al vs. NoAl) as the within-subject factor. For N2-NoGo, we executed a 2 × 2 design including group (BDs vs. CNs) as a between subject factor and type of stimuli (Al vs. NoAl) as a within-subject factor. (2) To test the hypothesized larger amplitude of P1 in response to Al stimuli in BDs, we used a 2 × 2 × 2 design, with group (BDs vs. CNs) as the between subject factor and with type of trial (Go vs. NoGo) and type of stimulus (Al vs. NoAl) as within-subject factors. Finally, we explored P3-Go only in Go trials, through a 2 × 2 design that included group (BDs vs. CNs) as a between subject factor and type of stimuli (Al vs. NoAl) as a within-subject factor. Bonferroni correction for multiple comparisons was applied on post hoc analysis. The alpha level was set at 0.05.

Behavioural performance was analysed across two variables related to reaction time (RT) (RT for correct responses to Go trials and RT for false alarms to NoGo trials) and two variables related to accuracy (percentage of hits for Go trials and percentage of correct omissions for NoGo trials). A 2 × 2 mixed-model ANCOVA was applied for each of these dependent variables, with group as a between-subject factor and type of stimulus as the within-subject factor.

Finally, we calculated the correlations between ERP components related to our hypothesis (P1, N2-NoGo, and P3-NoGo), behavioural performance data (RTs and accuracy), and measures related to the sample drinking characteristics (AUDIT scores, age of onset of drinking (AOD), total number of drinks in the last 180 days (TND), number of binge drinking episodes (BDEs) in the last 180 days, and BIS-11). Moreover, we used regression-based moderation analysis, implemented as model two in the process macro for SPSS (SPSS Inc., Chicago, IL, USA) [77] to assess the role of alcohol consumption intensity and history and its relationship with electrophysiological measures. These analyses were applied to those variables in which significant group differences were observed.

## 3. Results

### 3.1. ERP Components

The mean amplitudes (and standard deviations) of the selected ERP components are summarized in Table 4, according to the group (CN vs. BD) and to the type of trial and type of stimulus.

#### 3.1.1. P1 (TF06, PO8)

Analysis of the P1 amplitudes revealed significant main effects of the factors type of trial (F (1, 148) = 30.13, *p* < 0.001, η*p*^2^ = 0.169) (NoGo > Go) and type of stimulus (F (1, 148) = 40.33, *p* < 0.001, η*p*^2^ = 0.214) (Al > NoAl).

These main effects were, however, modulated by the interactions type of trial × type of stimulus (F (1, 148) = 15.51, *p* < 0.001, η*p*^2^ = 0.095) and group × type of trial × type of stimulus (F (1, 148) = 4.84, *p* = 0.029, η*p*^2^ = 0.032). Post hoc comparisons of the group × type of trial × type of stimulus interaction showed that, although in CNs the amplitude difference NoGo > Go remained significant for both Al (*p* = 0.044) and NoAl (*p* < 0.001) stimuli, in BDs this difference was only observed for NoAl (*p* < 0.001). In addition, the difference in P1 amplitude between stimuli (Al > NoAl) was only significant for Go trials in both CNs (*p* < 0.001) and BDs (*p* < 0.001).

#### 3.1.2. N2-NoGo (TF01, FCz)

**Figure 3:**
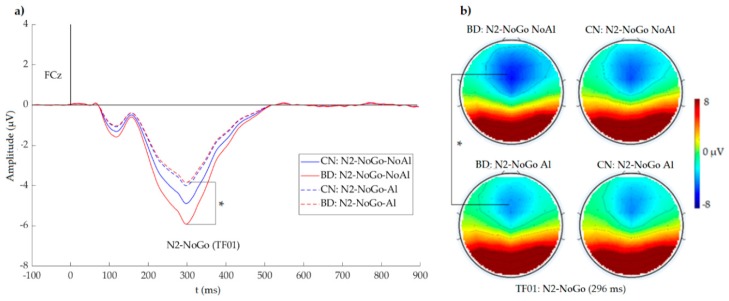
(**a**) N2-NoGo (TF01, FCz) waves for group (BD: continuous line, CN: dashed line) × type of stimulus (no alcohol: blue line, alcohol: red line) and (**b**) voltage topographical distributions at 296 ms. A group × type of stimulus interaction (*p* = 0.020) showed that the N2-NoGo amplitude was larger in response to NoAl than in response to Al stimuli (*p* < 0.0001) only in BD subjects.

In the main effect of type of stimulus (F (1, 148) = 20.29, *p* < 0.001, η*p*^2^ = 0.121), the amplitudes were larger for NoAl stimuli (more negative) than for Al stimuli (−4.4 vs. −3.1 µV). A significant group × type of stimulus interaction (F (1, 148) = 5.49, *p* = 0.020, η*p*^2^ = 0.036) was also observed. In the BDs, the N2-NoGo amplitudes were larger for NoAl than for Al (*p* < 0.001). No between-stimuli differences were observed in CNs (Figure 3).

#### 3.1.3. P3-NoGo (TF02, Pz)

Analysis of P3-NoGo revealed the main effects of type of stimulus (F (1, 146) = 18.314, *p* < 0.001, η*p*^2^ = 0.111) (NoAl > Al) and sex (F (1, 146) = 3.93, *p* = 0.049, η*p*^2^ = 0.026) (women > men). Interactions between sex × type of stimulus (F (1, 146) = 4.30, *p* = 0.040, η*p*^2^ = 0.029) and between group × sex × type of stimulus (F (1, 146) = 4.048, *p* = 0.046, η*p*^2^ = 0.027) were also observed. Post hoc analysis showed that, for the sex × type of stimulus interaction, NoAl > Al differences were only significant in women. For the group × sex × type of stimulus interaction, although in the CNs the NoAl > Al difference was significant in both women (*p* = 0.011) and men (*p* = 0.009), in BDs the corresponding difference was only significant in women (*p* < 0.001).

#### 3.1.4. P3-Go (TF04, Pz)

For P3-Go, only a main effect of type of stimulus (F (1, 148) = 15.47 *p* < 0.001, η*p*^2^ = 0.095) was observed; the P3-Go amplitude was larger for NoAl than for Al stimuli (5.54 vs. 4.83 µV). No other significant main effects or interactions were observed.

### 3.2. Behavioural Performance

The behavioural performance of the Go/NoGo task is summarized in Table 5, including the mean values and standard deviation for each group based on type of trial and type of stimulus.

#### 3.2.1. Reaction Time

No significant main effects or interactions were found for RT of hits. Type of stimulus had a main effect on the RT for false alarms (F (1, 148) = 18.536, *p* < 0.001, η*p*^2^ = 0.111); RT was slower for Al stimuli than for NoAl stimuli (439.04 ms vs. 409.29 ms). The cannabis covariate also had a main effect (F (1, 148) = 7.148, *p* = 0.008, η*p*^2^ = 0.046).

#### 3.2.2. Accuracy

Analysis of the percentage of hits (% hits) revealed a main effect of the type of stimulus factor on the accuracy of Go trials (F (1, 148) = 150.08, *p* < 0.001, η*p*^2^ = 0.503): the percentage of correct answers was higher for Al than for NoAl stimuli (98.22% vs. 94.43%) An interaction between type of stimulus × cannabis consumption covariate was also observed (F (1, 148) = 4.09, *p* = 0.045, η*p*^2^ = 0.027). Analysis of the percentage of correct omissions showed a main effect of type of stimulus (F (1, 148) = 4.19, *p* = 0.042, η*p*^2^ = 0.028), with greater accuracy in response to Al stimuli than in response to NoAl stimuli (88.17% vs. 86.68%).

### 3.3. Complementary Analysis

#### 3.3.1. Correlations Between Alcohol Consumption and Electrophysiological Variables

In BDs only, the amplitude of N2-NoGo in response to NoAl was correlated with the number of BDEs in the last 180 days (r = −0.303, *p* = 0.01) and with the total number of drinks in the last 180 days (r = −0.269, *p* = 0.023), and the amplitude of N2-NoGo in response to NoAl (in a context of predominant Al stimuli) was larger (more negative) in those subjects who reported more BDEs and consumption of a larger number of drinks. Age of onset of drinking was correlated with the P1 component, specifically the P1 elicited in Go-Al (r = −0.245, *p* = 0.039) and NoGo-NoAl trials (r = −0.240, *p* = 0.044). Early onset of drinking alcohol was correlated with larger P1 amplitudes in the context of preponderant Al stimuli. The P3-NoGo and P3-Go amplitudes were not significantly correlated with alcohol consumption variables.

#### 3.3.2. Behavioural Performance and Alcohol Consumption Correlations

Among BD subjects, the number of BDEs in the last 180 days correlated with the RT of false alarms to alcohol stimuli (r = −0.246, *p* = 0.039). Those subjects who reported a higher number of BDEs committed faster false alarms. There were no significant correlations between alcohol consumption variables and accuracy or RT of hits.

#### 3.3.3. Behavioural Performance and Electrophysiological Correlations

In BDs only, N2-NoGo in response to NoAl was correlated with RT to NoGo-NoAl (false alarms) (r = 0.343, *p* = 0.003), i.e., N2-NoGo amplitudes were larger in response to NoAl in those subjects who committed false alarms more quickly (or more impulsively). There were no other significant correlations between behavioural performance and electrophysiological variables

#### 3.3.4. Regression-Based Moderation Analysis

**Figure 4:**
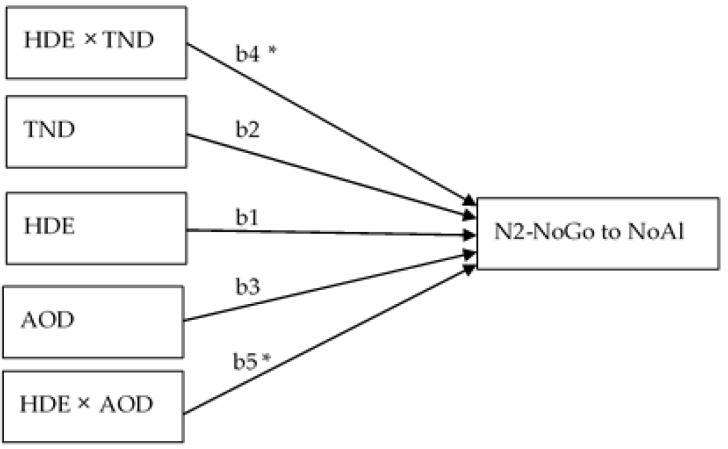
Diagram of the moderation model (model 2 from process): The two moderator variables considered in the model (total number of drinks in the last 180 days (TND) and age of onset of drinking (AOD)) interacted with the predictor variable (BDE) to explain the relation with the dependent variable (N2 NoGo to NoAl stimuli amplitude). BDE: binge drinking episodes in the last 180 days; TND: total number of drinks in the last 180 days; AOD: age of onset of drinking; b: regression coefficients; * *p* < 0.05.

Regression-based moderation analysis was executed for N2-NoGo elicited in response to NoAl. As alcohol consumption variables were defined as predictors and moderators, abstinent subjects (with no AOD reported) were excluded from this analysis. As a result, the model included 125 of the initial 151 subjects. Moderation analysis revealed that age of onset of drinking and the total number of drinks in the last 180 days acted as moderator variables in the association between binge drinking episodes (BDEs) and the N2-NoGo amplitude in response to NoAl. The moderation model, described in Figure 4, accounted for 16% of the variance of N2-NoGo in response to NoAl (R^2^ = 0.160, F (6,118) = 3.75, *p* = 0.0019).

The number of BDEs only significantly predicted N2-NoGo in response to NoAl amplitude when these BDEs were moderated by the total number of drinks in the last 180 days (intensity) or by the age of onset of drinking (history of consumption) (Figure 4). The regression coefficients for each variable and interactions are shown below (Table 6).

The change in R^2^ due to the interactions was significant for interactions between BDE and TND (F (1, 118) = 5.111, *p* = 0.026) and between BDE and AOD (F (1, 118) = 5.763, *p* = 0.018). The conditional effect of the number of BDE on the N2-NoGo amplitude was significant when it interacted with a larger total number of drinks in the last 180 days and later age of onset of drinking alcohol (b = −0.264, SE = 0.113, t = –2,3293, *p* = 0.0215). The highest values of N2-NoGo (more negative) related to BDEs were obtained in those subjects who consumed more alcohol in the last six months and whose age of onset of drinking was above the mean.

Finally, in order to provide a comprehensive overview of the main significant results of this research, a summary of the differences found between BD and CN is presented in Table 7.

## 4. Discussion

Dual-process models have been consistent in their description of severe addictive disorders (such as AUD) [13,18,78]. However, evidence in preclinical populations (e.g., in adolescent BDs without AUD) remains scarce. From a neurodevelopmental perspective, these models have described late adolescence as a period characterised by increased sensation seeking and reduced cognitive inhibitory control [11]. In this research, we aimed to investigate within the framework of dual-process models the extent to which binge drinking may influence the balance between the reflective and affective-automatic systems in a sample of first-year university students. To this end, the ERPs elicited during a beverage Go/NoGo task were recorded in order to explore the degree to which alcohol-related stimuli may modulate inhibition of a prepotent response in BDs without AUD.

According to our first hypothesis, we expected larger amplitudes of ERP components associated with conflict monitoring and response inhibition (N2-NoGo and/or P3-NoGo) in BDs. Consistently, in our experimental task, the N2-NoGo amplitude was larger in BDs (and not in CNs) in the NoGo-NoAl condition relative to the NoGo-Al condition. Moreover, regarding P3-NoGo, a gender/sex-related effect was observed, as there were no differences between the amplitudes of this component in response to Al and NoAl stimuli in male BDs.

### 4.1. BDs Displayed Increased Conflict Monitoring Activity in a Context of Frequent Al Stimuli

N2-NoGo is considered an index of detection of conflict between competing responses (Go and NoGo), with neural sources located in the IFC [36,37] and ACC [38,39]. Following the conflict monitoring theory of Botvinick, Cohen, and Carter [79], ACC acts as an online indicator of the degree of response conflict, conveying this information to other systems (such as the prefrontal cortex, including IFC) devoted more directly in the implementation of cognitive control strategies (attentional selection, response priming, goal maintenance, etc.). Consequently, N2-NoGo can be interpreted as an indicator of the previous cognitive processes required to implement inhibitory control rather than the actual inhibitory brake.

According to our N2-NoGo findings and conflict monitoring theory, it is possible that, in BDs, the Al stimuli may enhance activation of the prepotent Go pre-response so that the conflict will thus increase during NoGo trials. BDs would therefore have to expend greater cognitive effort and to recruit extra neural resources to successfully inhibit the primed Go response. In terms of the dual-process model framework, during the Go-Al vs. NoGo-NoAl condition, BDs may display overactivation of the automatic system (due to the higher motivational value of Go-Al stimuli). Extra neural resources may be allocated to the reflective system (as reflected by larger N2-NoGo amplitudes) to overcome this overactivation and to successfully resolve the conflict.

Previous neurocognitive research on binge drinking has already shown that alcohol-related content may modulate N2-NoGo amplitudes of prepotent response inhibition. As we discuss below, the direction and intensity of these modulations differ between studies, possibly due to methodological factors, such as different task designs or sample characteristics. In those studies reporting greater activation in the absence of behavioural differences, the results were interpreted within the context of the compensatory hypothesis.

In an fMRI design, Ames et al. [51] observed greater activity in the right dorsolateral and medial prefrontal cortex, anterior cingulate cortex, and anterior insula in BDs during NoGo-Al trials of a beverage Go/NoGo task. These authors suggested that, in BDs, the increased incentive value or salience of the alcohol pictures during NoGo trials may have served as an attentional bias cue, resulting in greater effort being required to withhold a response. Although the task only included the Go-NoAl vs. NoGo-Al condition under a different design and therefore we cannot fully compare their results with ours, the increased activation also indicates compensatory activity of neural regions related to N2-NoGo (medial prefrontal cortex and ACC).

In a Go/NoGo task with geometrical shapes used as Go stimuli and a combination of other geometrical shapes, Al pictures, and NoAl pictures as NoGo stimuli, Watson et al. [47] found that the NoAl stimuli generally elicited larger N2-NoGo than the Al stimuli. However, in those subjects who reported more episodes of intoxication in the last six months, the N2-NoGo amplitude was larger in response to Al stimuli. This difference relative to our findings may be associated with the task design, as the Go trials consisted entirely of nonbeverage, neutral stimuli so that conflict may emerge in a different way.

Task design may also explain the absence of N2-NoGo differences in a letter Go/NoGo task with pictures of alcoholic beverages and neutral pictures as a background [48]. The fact that alcohol pictures were not explicitly related to task instructions may have led to the lack of differences, as shown by Lannoy et al. [49] in a study in which group differences in N2-NoGo amplitudes and behavioural performance only emerged when Al pictures were explicitly related to the task instructions. Furthermore, in contrast to the compensatory hypothesis, the aforementioned research found smaller N2-NoGo amplitudes for Al pictures in BDs (relative to CNs), suggesting that BDs may experience some difficulties at the attentional level in inhibiting a prepotent response when alcohol pictures are present. In comparison with our study, the level of alcohol consumption (mean AUDIT score: 17.20 ± 5.32) in the previous sample may suggest a more advanced state of deleterious cerebral effects as a result of a higher rate of consumption. It is therefore possible that the compensatory mechanisms that may be acting in our sample may have been unavailable in the subjects of the previous research. A follow-up study of our sample would be necessary to confirm this explanation.

Considering our findings, the amplitude of N2-NoGo elicited in response to NoAl stimuli also appeared to be related to the rate of alcohol consumption. Correlational and regression-based moderation analyses revealed that N2-NoGo in response to NoAl was significantly correlated with the number of BDEs in the last six months and with the total number of alcoholic drinks in the last six months. These correlations showed that, in BDs, more BDEs and a greater number of drinks were related to larger amplitudes of this ERP component. Regression-based moderation analysis showed that, in this group, the amplitude of N2-NoGo in response to NoAl was predicted by the number of BDEs specifically when this variable interacted with the total amount of drinks and with the age of onset of drinking. In other words, N2-NoGo amplitudes were largest in subjects with a short history of binge alcohol consumption but with a high frequency and intensity.

### 4.2. Male BDs Did Not Show Differences in Evaluation/Closure of the Inhibition Process Between Alcohol and Nonalcohol Stimuli

P3-NoGo has been related to closure or the evaluation of inhibition process [43], and its neural source has been located in IFC [36]. Results showed a group × sex × type of stimulus interaction regarding the amplitude of this component. During a context of predominant Al stimuli, NoGo-NoAl trials elicit larger P3-NoGo, but this difference was not significant in male BDs. It seems that, in male BDs, the evaluation/closure of the inhibition process may be altered by the motivational value of the stimuli.

To our knowledge, no gender/sex-related differences in P3-NoGo have previously been reported in research on binge drinking. Gender/sex differences related to BD in young samples have been explained in terms of neurodevelopmental delay in men relative to women [80], together with differences in alcohol consumption patterns, expectations, and effects [81]. Nevertheless, replication of these results and longitudinal data are required to further clarify these gender/sex-related differences and how they evolve over time.

Regardless of gender/sex differences and alcohol-related content, previous electrophysiological research with neutral Go/NoGo tasks has detected P3-NoGo compensatory alterations related to binge drinking. A longitudinal study by our research group reported larger P3-NoGo amplitudes (in response to neutral stimuli) in BDs than in CNs, together with greater activation of the right inferior frontal cortex, only at the second evaluation (20–21 years old and at least two years of binge drinking) [55]. As there are great similarities between the sample used in the aforementioned study during the first evaluation and the sample of our study, it is possible that anomalies in P3-NoGo amplitude will emerge in subsequent follow-ups of our sample if the binge drinking pattern persists.

### 4.3. Increased Salience of Al Stimuli.

P100 is an early ERP component that is sensitive to variations in stimulus parameters but that also can be modulated by attentional influences [54]. Our second hypothesis posits that greater salience of Al stimuli for BDs will be reflected by larger amplitude of the P1 component. The results showed that Al stimuli elicited larger P1 amplitudes; however, no differences in relation to groups were found, except for a second-order interaction (group × type of trial × type of stimulus*)*. Post hoc analysis did not reveal any group-based differences in P1 amplitudes, so at least in our sample, BDs did not show increased responses to alcohol pictures at the perceptual level.

Interestingly, correlational analysis showed that an early onset of drinking was correlated with larger P1 amplitudes in the context of prepotent Al stimuli. This is consistent with previous electrophysiological research (in oddball tasks) showing increased P1 amplitudes to Al stimuli in BDs [51,52]. However, these findings are not consistent with those of a longitudinal study in which a reduction in P1 amplitude was observed in response to both Al and NoAl stimuli after only one year of a binge drinking pattern of consumption [82]. This incongruence between the findings of both studies may be due to aspects of sample selection, such as the inclusion, in the longitudinal research [82], of subjects with family history of alcohol consumption. Further replication of these results from a longitudinal approach is required.

### 4.4. An Integrated View of Beverage Go/NoGo Tasks Through the Cognitive Stream

From all of the above, the findings related to the modulations of response inhibition by motivational (alcohol-related) stimuli in BDs seem to indicate anomalies in ERP components at different stages of the cognitive stream, which may depend on the history and intensity of binge drinking. In early perceptual components, the scarce findings range from subtle correlations (as in our research) to larger [52,53] or smaller [82] P1 amplitudes. Regarding later cognitive ERP components, larger N2-NoGo amplitudes in response to nonalcohol stimuli (as we observed) may reflect compensatory conflict monitoring processes that overcome bias to an alcohol preponderant context; however, such compensatory mechanisms may be overcome by a longer history of consumption (or more difficult task), as reflected by lower N2-NoGo or poorer behavioural execution reported in other studies [45,49]. The same seems to occur with the P3-NoGo component, as larger amplitudes were reported in a longitudinal research [55] only during the second evaluation. In summary, during the first years of a binge drinking pattern of consumption, compensatory mechanisms still seem to be available in fairly simple inhibitory tasks and Al stimuli seem to affect conflict monitoring rather than inhibitory processes per se. Longitudinal data are needed to confirm whether this compensatory mechanism will no longer be available if the binge drinking pattern is maintained as well as to explore whether alcohol bias or response inhibition alterations will occur.

### 4.5. Limitations and Future Directions

Some limitations and future directions from the present study must be acknowledged. First, although the picture set was carefully designed according to ABPS characteristics [69], it has not yet been validated. Secondly, it has been suggested that nonalcohol pictures may also have a motivational value and that more neutral (not beverage-related) pictures would provide better control of stimulus salience. Third, the sample characteristics (university students) do not allow generalization of the results to other populations. Future research should address the need for longitudinal designs to explore the evolution and causality of the alterations related to binge drinking. Finally, the use more of challenging task designs in these preclinical samples would enable better exploration of behavioural data and error-related processes.

## 5. Conclusions

In summary, the findings of the present study revealed that, in a sample of first-year university students, the conflict monitoring system of binge drinkers (relative to controls) is sensitive to the alcohol and nonalcohol content of pictures presented during a beverage Go/NoGo task, as manifested by anomalies in ERPs. Specifically, a larger amplitude of the frontocentral N2-NoGo component elicited in response to NoAl images (in comparison with N2-NoGo in response to Al images) was found in BDs in the absence of differences in behavioural performance. According to dual-process models, it seems that a context of predominant Al stimuli may enhance the preponderance of the Go response through overactivation of the affective-automatic system. BDs may be able to successfully inhibit this prepotent response by means of compensatory neural activity of the reflective system so that no other important ERP anomalies should be found in relation to response inhibition.

Future research is needed to replicate and extend the current findings, specifically to disentangle possible gender/sex modulations and the consequences of a longer history of binge drinking (such as the emergence of an inhibitory impairment and its interaction with motivational bias to alcohol cues). Longitudinal designs would be particularly appropriate in this respect.

In light of the present results and previously reported findings, the dual-process model of addictive disorders in adolescents is an appropriate comprehensive framework for exploring the consequences and implications of binge drinking, although future refinements or even new formulations of this model [44] could be incorporated. Under this perspective, neurocognitive programmes that encompass cognitive abilities (such as conflict monitoring and inhibition) and also the influence of motivational/affective processes (such as alcohol-related bias) could be developed. Research on this type of intervention remains scarce and necessary but has already shown promising results in reducing alcohol consumption in heavy drinking young adults [83].

## Figures and Tables

**Table 1:** Inclusion and exclusion criteria, based on interview.

**Inclusion Criteria**	**Exclusion Criteria**
Alcohol and cannabis consumption: ∙ Control group (CN): Alcohol consumption < 6 BDE ^1^ over the last 180 days (6 months) and cannabis consumption < 12 units over the last 90 days (3 months) ^2^ ∙ Binge drinking group (BD): Alcohol consumption ≥ 6 BDE over the last 180 days and cannabis consumption < 12 units over the last 90 days ^2^	∙ Chronic pathologies that could affect neurocognitive functioning (hypothyroidism, diabetes, liver diseases, etc.) ∙ History of neurological disorder or history of brain injury with loss of consciousness for longer than 20 min ∙ History of diagnosed psychopathological disorders (axis I and II, according to (DSM-IV-TR) criteria) ∙ SCL-90-R score > 90th percentile on Global Severity Index (GSI) or ≥2 symptoms dimensions. ∙ Family history of major psychopathological disorders in first-degree relatives ∙ Family history of first-degree alcoholism or substance use disorder ∙ Regular consumption of drugs with psychoactive effects (psycholeptics) ∙ Use of illegal drugs (except cannabis) in the last 6 months ∙ Non-corrected sensory or motor deficits that could prevent subjects from a normal execution on experimental tasks

^1^ A binge drinking episode (BDE) was defined as the consumption of 5/7 (females/males) standard drinking units (SDU; 10 g of alcohol, according to Spanish definition of the SDU) on one occasion, raising blood alcohol concentration above 0.08 g/dl [2]. ^2^ Subjects who regularly consumed cannabis (1 or more units per week, e.g., References [66,67]) were not included.

**Table 2:** Sociodemographic and drinking characteristics of the Control and BD groups with mean (SD): Significant differences between groups are indicated according to the p level.

Sociodemographic and Drinking Variables	Control	Binge Drinkers
*n*	80	71
Gender (male/female)	42/38	28/43
Age	18–19	18–19
Tobacco consumption (>2 cigarettes/day) ^1^	0	5
Cannabis consumption (>3 and <12 in the last 90 days) ^2^	1	11
SCL-90-R: GSI (percentile scores) *	44.5 (26.71)	57.41 (23.52)
Barrat Impulsivity Scale 11 (BIS-11) ** ∙ Cognitive ∙ Motor ** ∙ Unplanned	59.95 (8.64)17.86 (3.46)19.04 (4.16)23.05 (0.58)	64.66 (8.12)18.87 (2.98)21.61 (4.08)24.18 (4.99)
Age of onset of drinking **	16.24 (1.13)	15.42 (1.14)
BDE (last 180 days) **	0.71 (1.54)	20.99 (10.70)
Nº drinks during peak consumption (2 h lapse) **	2.24 (1.29)	5.01 (1.38)
AUDIT score **	1.99 (2.71)	9.55 (4.85)

^1^ Maximum 6 cigarettes per day: none of the participants met the criteria for nicotine dependence, according to the Nicotine Dependence Syndrome Scale, short version (NDSS-S) scores [62]. ^2^ None of the participants met the criteria for problematic cannabis use, according to the Observatorio Español De Las Drogas y las Adicciones (OEDA) criteria based on Cannabis Abuse Screening Test (CAST) scores [68]. * *p* < 0.05; ** *p* < 0.001. BDE = Binge drinking episodes. SCL-90-R: GSI = Symptom Checklist-90-Revised: Global Severity Index. AUDIT = Alcohol Use Disorders Identification Test.

**Table 3:** Temporal PCA factors: All of the factors identified are ordered by the explained variance, with their latency (ms) and maximum negative and positive locations (absolute maximum in bold). The factors with the event-related potential (ERP) components of interest for the study are included in the final column.

Factor	PeakLatency (ms)	Peak (–) Channel	Peak (+) Channel	Variance	Unique Variance	ERP Wave Identified
TF01	296	FCz	PO8	0.397	0.164	N2
TF02	532	FP1	Pz	0.214	0.0887	P3-NoGo
TF03	844	PO7	CPz	0.112	0.0685	-
TF04	416	F8	Pz	0.084	0.024	P3-Go
TF05	156	Fp2	PO3	0.036	0.016	
TF06	100	Fp1	PO8	0.027	0.009	P1
TF07	232	Oz	PO8	0.023	0.011	-
TF08	124	FC3	PO8	0.021	0.013	
TF09	336	PO8	F8	0.017	0.012	-
TF10	892	PO8	CPz	0.015	0.008	-

**Table 4:** Mean (SD) amplitude (μV) of each ERP component for different groups (control and binge drinkers) and type of stimulus (alcohol vs. nonalcohol; Go vs. NoGo).

Type of Stimulus	Group	P1	N2-NoGo	P3-NoGo	P3-Go
NoGo-NoAl	Control	3.80 (3.40)	–3.89 (4.54)	12.99 (5.42)	--
	Binge drinkers	4.39 (3.59)	–4.88 (4.52)	13.54 (5.54)	--
NoGo-Al	Control	4.26 (3.59)	–3.24 (4.14)	10.81 (5.83)	--
	Binge drinkers	4.25 (3.56)	–2.97 (4.78)	11.28 (5.47)	--
Go-NoAl	Control	2.90 (2.93)	--	--	5.72 (3.53)
	Binge drinkers	3.08 (3.12)	--	--	5.34 (4.03)
Go-Al	Control	3.77 (2.86)	--	--	4.74 (3.45)
	Binge drinkers	4.33 (3.49)	--	--	4.91 (3.72)

**Table 5:** Mean (SD) reaction times (on hits and false alarms (FA) in milliseconds (ms)) and accuracy (% of hits and correct omissions) for each group. There were no significant differences in behavioural performance between groups.

Variable	Type of Stimulus	Controls	Binge Drinkers
Reaction time (ms)	Go-NoAl	505.83 (59.08)	506.38 (70.72)
	Go-Al	509.05 (59.60)	506.36 (66.21)
	NoGo-NoAl (FA)	422.79 (62.98)	395.38 (59.20)
	NoGo-Al (FA)	447.42 (85.51)	429.77 (79.55)
% Correct responses (Go) and omissions (NoGo)	Go-NoAl	94.43 (3.36)	95.55 (3.42)
	Go-Al	98.56 (1.73)	98.48 (2.00)
	NoGo-NoAl	86.13 (11.42)	87.23 (12.04)
	NoGo-Al	89.08 (9.12)	87.23 (11.28)

**Table 6:** Regression coefficients from the moderation analysis: Slope of the linear regression (b), standard error (SE), and t statistic. The interactions between the predictor (BDE) and the two moderator variables (TND and AOD) were significant. * *p* < 0.05.

Variable	b	SE	t	*p*
BDE	−0.106	0.087	−1.221	0.224
TND	0.005	0.008	0.639	0.524
AOD	0.442	0.340	1.299	0.196
BDE × TND *	−0.001	0.001	−2.261	0.026
BDE × AOD *	−0.070	0.0292	−2.401	0.018

**Table 7:** Summary of the results where effects of the group factor (control vs. binge drinkers) or interactions with type of trial (Go vs. NoGo) or type of stimulus (Al vs. NoAl) were significant. The associated p-values are shown in parentheses; n.s.: no significant.

Type of Analysis/Variable	Controls	Binge Drinkers
**ANCOVA**		
P1	Al: NoGo > Go (*p* < 0.044)	Al: Go = NoGo (n.s.)
N2_NoGo	NoAl = Al (n.s.)	NoAl > Al (*p* < 0.001)
P3_NoGo	Males: NoAl > Al (*p* = 0.009)	Males: NoAl = Al (n.s.)
**Correlations**		
P1/Age of onset of drinking	Go-Al (n.s.)	Go-Al (r = −0.245, *p* = 0.039)
	NoGo-NoAl (n.s.)	NoGo-NoAl (r = −0.240, *p* = 0.044)
N2-NoGo/No BDEs	NoAl (n.s.)	NoAl (r = −0.303, *p* = 0.010)
N2-NoGo/TND	NoAl (n.s.)	NoAl (r = −0.269, *p* = 0.023)
RT false alarms/N^o^ BDEs	NoAl (n.s.)	NoAl (r = −0.246, *p* = 0.039)
N2-NoGo/RT false alarms	NoAl (n.s.)	NoAl (r = 0.343, *p* = 0.003)
